# Naringenin Induces Pathogen Resistance Against *Pseudomonas syringae* Through the Activation of NPR1 in *Arabidopsis*

**DOI:** 10.3389/fpls.2021.672552

**Published:** 2021-05-20

**Authors:** Jonguk An, Sun Ho Kim, Sunghwa Bahk, Uyen Thi Vuong, Nhan Thi Nguyen, Huy Loc Do, Sang Hee Kim, Woo Sik Chung

**Affiliations:** Division of Applied Life Science (BK21 Plus Program), Plant Molecular Biology and Biotechnology Research Center, Gyeongsang National University, Jinju, South Korea

**Keywords:** flavonoid, MAPK, naringenin, NPR1, pathogen resistance, PR1, SA

## Abstract

Flavonoids are well known for the coloration of plant organs to protect UV and ROS and to attract pollinators as well. Flavonoids also play roles in many aspects of physiological processes including pathogen resistance. However, the molecular mechanism to explain how flavonoids play roles in pathogen resistance was not extensively studied. In this study, we investigated how naringenin, the first intermediate molecule of the flavonoid biosynthesis, functions as an activator of pathogen resistances. The transcript levels of two *pathogenesis-related (PR)* genes were increased by the treatment with naringenin in *Arabidopsis*. Interestingly, we found that naringenin triggers the monomerization and nuclear translocation of non-expressor of pathogenesis-related genes 1 (NPR1) that is a transcriptional coactivator of *PR* gene expression. Naringenin can induce the accumulation of salicylic acid (SA) that is required for the monomerization of NPR1. Furthermore, naringenin activates MPK6 and MPK3 in ROS-dependent, but SA-independent manners. By using a MEK inhibitor, we showed that the activation of a MAPK cascade by naringenin is also required for the monomerization of NPR1. These results suggest that the pathogen resistance by naringenin is mediated by the MAPK- and SA-dependent activation of NPR1 in *Arabidopsis*.

## Introduction

Flavonoids are secondary metabolites widely distributed in plants. Flavonoids can be divided into several subgroups by the diversity of chemical groups. Flavonoids have roles in many facets of plant physiology (Buer et al., 2010). Major roles of flavonoids are UV protectants (Shirley, 1996), antioxidants and scavengers of reactive oxygen species (Rice-Evans, 2001). The other roles of flavonoids include pollinator attractants (Mol et al., 1998), root nodulation (Mandal et al., 2017), allelopathy (Hassan and Mathesius, 2012) and auxin transport inhibitor (Peer and Murphy, 2007). The previous study revealed that flavonoids also act in resistance against pathogens and herbivores (Treutter, 2005). Naringenin is one of the major flavonoids which was broadly distributed in citrus fruits and vegetables such as grapefruit, lemon, oranges and tomatoes (Manchope et al., 2017). Naringenin is accumulated by infected biotrophic pathogen *Plasmodiophora brassicae* and *Pseudomonas siringae pv. pisi* (Päsold et al., 2010; Makarova et al., 2016). Furthermore, naringenin is reported to confer not only anti-inflammatory and antiviral activities (Hartogh and Tsiani, 2019) but also resistance against *Fusarium* (Skadhauge et al., 1997) and *Pyricularia oryzae* (Padmavati et al., 1997). However, the pathway to explain how naringenin activates pathogen resistance has not been investigated.

Salicylic acid (SA) mediates plant defense against biotrophic and hemibiotrophic pathogens. SA is accumulated in both infected and distal leaves in response to pathogen attack (van Butselaar and Van den Ackerveken, 2020). In Arabidopsis, SA biosynthesis is produced primarily through the isochorismate pathway and the phenylalanine ammonia-lyase pathways (Ding and Ding, 2020). SA-mediated immune responses are essential parts of both PTI and ETI (Tsuda et al., 2009). SA is required for the expression of *pathogenesis-related* genes and the synthesis of defensive compounds associated with both local and systemic acquired resistance in plants (An and Mou, 2011). In Arabidopsis, the exogenous application of SA suffices to establish SAR that evokes enhanced basic resistance to a variety of pathogens (Klessig et al., 2018).

The NPR1, NPR3 and NPR4 bind to SA and function as SA receptors. NPR1 functions as a transcriptional coactivator to induce *PR* gene expression, whereas NPR3 and NPR4 function as transcriptional co-repressors to repress *PR* gene expression (Fu et al., 2012; Ding et al., 2018). By the binding of SA, NPR1 is activated, whereas NPR3 and NPR4 are inactivated. NPR1 activated by SA plays a critical role in resistance against biotrophic pathogen challenge (Cao et al., 1997). In the absence of SA, NPR1 is found as oligomer forms in the cytoplasm. By the accumulation of SA, changes in cellular redox potential lead to the reduction of NPR1 through the activity of thioredoxins. This reduction of NPR1 contributes to the monomerization of NPR1 (Mou et al., 2003; Tada et al., 2008). The monomerized NPR1 is translocated from the cytosol into the nucleus via a bipartite nuclear localization signal (Kinkema et al., 2000; Maier et al., 2011). The nuclear-localized monomeric NPR1 interacts with TGA transcription factor to induce *PR* gene expression (Zhang et al., 1999; Kim and Delaney, 2002).

Mitogen-activated protein kinases (MAP kinase) are highly conserved serine/threonine-specific protein kinases that respond to various extra- and intracellular signals in all eukaryotes (Meng and Zhang, 2013). A basic MAPK cascade consists of three distinct kinases. MAP kinase kinase kinases (MAPKKK) receive signals from upstream receptors and activate downstream MAP kinase kinases (MAPKK) via phosphorylation, which in turn phosphorylates and activates MAPK (Zhang et al., 2018). MAPK cascades play important roles in the earliest signaling events upon a perception of PAMPs, DAMPs or effectors (Bartels et al., 2013; Thulasi Devendrakumar et al., 2018). In response to pathogens, MPK3 and MPK6 positively contribute to innate immune responses in plants via phosphorylation of downstream substrates in a partially redundant manner in Arabidopsis (Beckers et al., 2009; Meng and Zhang, 2013; Xu et al., 2016). For example, MPK6 phosphorylates ACS6, an enzyme of ethylene biosynthesis, which increases ethylene production (Han et al., 2010). MPK3 phosphorylates and activates the transcription factor VIP1 and ERF6, which activates defense-related genes (Bethke et al., 2009). The transcription factor WRKY33 is directly phosphorylated by MPK3 and MPK6. WRKY33 is required for MPK3 and MPK6-mediated production of camalexin and pipecolic acid (Mao et al., 2011; Wang et al., 2018).

In this study, we investigated the molecular mechanism to explain how naringenin induces pathogen resistance. We showed that naringenin induces the monomerization and nuclear translocation of NPR1 that is triggered by both the accumulation of SA and the activation of MAPK cascade.

## Materials and Methods

### Plant Materials and Growth Conditions

The *Arabidopsis thaliana* ecotype Columbia-0 (Col-0) plants, *mapk3-2* (SALK_151594), *mapk6-3* (SALK_127507), *sid2* mutants, *NahG* and *35S:NPR1-GFP* in *npr1-2* were used. Seeds were surface sterilized with 70% EtOH and with 1/10-diluted commercial bleach (0.4% NaOCl) and washes with distilled water. Surface-sterilized seeds germinated on agar plates containing Murashige-Skoog (MS) salts and vitamins (Murashige and Skoog, 1962), 2.0% sucrose and 0.8% agar. The MS plates were kept for 3 d at 4°C in the dark, and then at 22°C in a growth chamber under a 16 h light/8 h dark photoperiod with a light intensity of ∼120 μmol m^–2^ s^–1^. For bacterial growth curve assays, 14-day-old seedlings were transplanted into soil and then grown under at 24°C in a growth chamber under an 8 h light/16 h dark photoperiod with 70% relative humidity and with a light intensity of ∼120 μmol m^–^^2^ s^–1^ for 14 days.

### Antimicrobial Activity

*Pseudomonas syringae* pv. *tomato* DC3000 (*Pst* DC3000) was incubated on Pseudomonas agar F (BD DIFCO, United States) with 30 mg/L rifampicin at 28°C for 48 h. Cells were resuspended in 10 mM MgCl_2_ to form a gradient concentration of 10^7^, 10^8^ and 10^9^ CFU ml^–1^. The bacterial suspensions were dropped and incubated on King’s B (KB) medium sprayed with 0, 0.5, 1, 2, and 4 mM naringenin and were incubated at 28°C for 24 h.

### Bacterial Growth Curve Assays

Growth curve assays with the virulent *Pst* DC3000 were performed as described previously (Gassmann, 2005). In brief, pre-sprayed leaves of four-week-old plants with 100 μM naringenin were sprayed with 2 × 10^8^ cfu/ml bacterial suspensions in 10 mM MgCl_2_ and 0.01% of Silwet L-77. At indicated time points, leaf discs (total area 1 cm^2^) were harvested from the infected tissues. The samples were ground in 10 mM MgCl_2_ and were plated in serial dilutions on selective plates. A two-tailed Student’s *t*-test was used for statistical analysis of bacterial growth in different plant lines.

### Total RNA Extraction and Quantitative PCR

Total RNA was isolated from 2-week-old plants infiltrated with 0.5 μM flg22 or 100 μM naringenin for 24 h using an RNA purification kit (Macherey-Nagel, Germany). RNA (5 μg) was reverse transcribed using SuperScript II RNase-Reverse Transcriptase (Invitrogen, United States). Quantitative PCR (qPCR) was performed in a 10 μl reaction volume containing 1 μl RT products, 10 pmol of gene-specific primers and 5 μl SsoFast EvaGreen Supermix (Bio-Rad, United States) using the CFX384 Real-Time System (Bio-Rad, United States). The reaction conditions included an initial 5 min pre-incubation at 94°C, 45 cycles of 94°C for 30 s, 55°C for 30 s and 72°C for 40 s, followed by melting curve analysis via 90 cycles at 55°C, increasing by 0.5°C/cycle and a final cooling step for 10 min at 72°C. The primers used for qPCR are shown in Supplementary Table 1.

### Protein Extraction and Nuclear Fractionation

Total proteins were extracted from two-week-old plant infiltrated with 0.5 μM flg22 or 100 μM naringenin in the absence or presence of 50 μM PD98059 (Sigma-Aldrich, United States) by grinding in liquid nitrogen and resuspending powder in protein extraction buffer (50 mM HEPES, pH 7.5, 5 mM EDTA, 5 mM EGTA, 1 mM Na_3_VO_4_, 25 mM NaF, 50 mM-glycerophosphate, 2 mM DTT, 2 mM PMSF, 5% glycerol, 1% Triton X-100 and protease inhibitor). After two rounds of centrifugation at 12,000 × g for 15 min, the supernatants were transferred to tubes and stored at −80°C until use. Protein concentrations of supernatant were determined using a Bio-Rad Protein Assay kit (Bio-Rad, United States). Nuclear fractionation was performed based on the previously described (Kinkema et al., 2000). Briefly, nuclear proteins were extracted from 2-week-old plants infiltrated with 0.5 μM flg22 or 100 μM naringenin for 3 h using CELLYTPN1 CelLytic PN Isolation/Extraction Kit (Sigma-Aldrich, United States).

### Immunoblot Analysis

For detection of NPR1 proteins, samples of total protein (30 μg) were resolved on 8% SDS-PAGE and transferred to a PVDF membrane (Bio-Rad, United States). Primary and secondary antibodies were used rabbit anti-NPR1 (1:5,000; Abiocode, United States) antibodies and horseradish peroxidase-conjugated anti-rabbit antibodies (1:10,000), respectively. Signals were visualized using an ECL kit (Bio-Rad, United States).

For detection of MPK activities, total protein (30 μg) were resolved on 10% SDS-PAGE and transferred to a PVDF membrane (Bio-Rad, United States). Primary and secondary antibodies were used rabbit anti-phospho-p42/44 MAPK (1:2,000, Cell Signaling Technology, United States) antibodies and horseradish peroxidase-conjugated anti-rabbit antibodies (1:10,000), respectively. Signals were visualized using an ECL kit.

For detection of nuclear and cytosol proteins, nuclear and cytosolic fractions were resolved by 10% SDS-PAGE. The proteins were detected with rabbit anti-NPR1 (1:5,000; Abiocode, United States), anti-H3 (1:5,000, Abcam, United Kingdom) and anti-PEPC (1:10,000, Abcam, United Kingdom) antibodies as primary antibodies and horseradish peroxidase-conjugated anti-rabbit (1:10,000) antibodies as secondary antibodies. Signals were visualized using an ECL kit.

### Confocal Microscopy

Arabidopsis leaf tissues were mounted in water and viewed with a confocal laser-scanning microscope (Olympus FV1000). GFP was visualized using an excitation wavelength of 488 nm nanometer beam splitter.

### Determination of Salicylic Acid

Two-week-old plants were infiltrated with naringenin and incubated for 24 h. Free SA was isolated and quantified as described previously (Pan et al., 2010). Ground tissue (50 mg) was used for free SA measurement. The ground sample was extracted with 500 μl of extraction solvent 2-propanol/H_2_O/concentrated HCl (2:1:0.002, v/v/v) containing d6-SA an internal standard for SA, respectively for 24 h at 4°C. Dichloromethane (1 mL) was added to the supernatant and then centrifuged at 13,000 g for 5 min at 4°C. The lower phase, which was taken into a clean screw-cap glass vial, was dried under nitrogen and resolved in pure methanol. Complete dissolved extract ensured by vortexing and sonicating was transferred to a reduced volume liquid chromatography vial. SA was analyzed by a reverse-phase C18 Gemini high-performance liquid chromatography (HPLC) column for HPLC electrospray ionization tandem mass spectrometry (HPLC–ESI–MS/MS) analysis. These experiments were repeated three times with similar results.

### DAB and NBT Staining

Two-week-old seedlings were infiltrated with 0.2 mM SA and 100 μM naringenin and were incubated for 24 h. To detect hydrogen peroxide, seedlings were submerged into 1 mg/ml DAB (Sigma, United States) solution for 6 h. The stained seedlings were transferred to EtOH and incubated at 70°C for 10 min to remove chlorophyll. To detect superoxide anion, seedlings were submerged into 1 mM NBT (Sigma, United States) with 10 mM sodium azide in 50 mM sodium phosphate buffer (pH 7.4) for 24 h. The stained seedlings were transferred to EtOH and incubated to remove chlorophyll. All materials were visualized using a light microscope.

### Plasmid Construction and Expression of Recombinant Proteins

Plasmid construction was performed as previously described with minor modification (Kim et al., 2017). For the construction of the GST-SnRK2.8, the SnRK2.8 was amplified by PCR with gene-specific primers (Supplementary Table 2). The cDNA PCR fragment was cloned into the T-blunt vectors (Solgent, Korea) and their accuracy was verified by sequencing. To create in-frame N-terminal GST fusions, the inserts were excised with *Bam*HI and *Xho*I and cloned into the pGEX 4T-1 vector (Amersham Biosciences, United States). The GST-fusion constructs were transformed into BL21 (DE3) *E. coli* and GST fusion proteins were expressed and purified using glutathione Sepharose-4B beads according to the manufacturer’s instructions (GE Healthcare, United States). For the construction of His-MAPKs, full-length MPK3, MPK4 or MPK6 were amplified by PCR with gene-specific primers (Supplementary Table 2). The cDNA PCR fragment was cloned into the T-blunt vectors and their accuracy was verified by sequencing. To create in-frame His fusion, the inserts were excised with *Bam*HI and *Sal*I, and subcloned into pQE30 or pGEX 4T-1 vector. The His-fusion constructs were transformed into *E. coli* (M15) and His-tag fusion proteins were expressed and purified using Ni-NTA agarose beads according to the manufacturer’s instructions (Qiagen, Germany).

### *In vitro* Kinase Assays

*In vitro* kinase reactions were performed as previously described with minor modification (Liu and Zhang, 2004). In brief, recombinant kinases and substrates proteins were mixed in kinase reaction buffer (25 mM Tris-HCl [pH 7.5], 1 mM DTT, 20 mM MgCl_2_, 2 mM MnCl_2_, and 50 μM [γ-^32^P] ATP). His-MAPKs (1 μg) were used as kinases. GST (1 μg; negative control), myelin basic protein (MBP, 0.5 μg; positive control), and GST-SnRK2.8 variants (2 μg) were used as substrates. The reactions were begun using 1 μCi [γ -^32^P] ATP at 30°C for 30 min. The kinase reactions were stopped by adding SDS sample buffer and boiling for 5 min. Reaction products were resolved by 10% SDS-PAGE. The gels were autoradiographed and stained with Coomassie Brilliant Blue (CBB) R-250, using pre-stained markers to estimate protein size.

## Results

### Naringenin Enhances the Resistance to *Pst* DC3000

**FIGURE 1:**
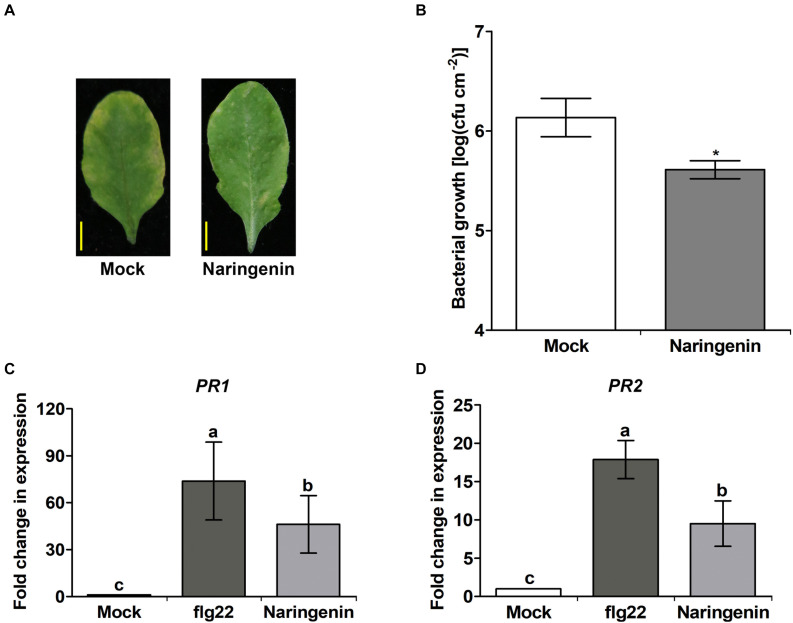
Basal resistance to *Pst* DC3000 infection of Col-0 plants is enhanced in the presence of naringenin. The growth of *Pst* DC3000 in control (mock) or in naringenin-pretreated Col-0 plants. Col-0 plants were sprayed with 1 mM naringenin one day before being challenged with DC3000. **(A)** Disease symptoms of Col-0 plants infected by *Pst* DC3000 in the absence and presence of naringenin. Photos were taken at three days. **(B)** Bacterial numbers were quantified three days later. Bars represent mean values (SD) of colony-forming units (cfu) per square centimeter from biological replicate samples derived from different plants. Each biological replicate consists of three leaf disks harvested from different leaves of one plant. Number signs denote statistically significant differences from the Col-0 value (*P < 0.05; two-tailed *t*-test). Scale bar represents 0.5 cm. The expressions of *PR* genes are induced by naringenin. Total RNA was extracted from Col-0 plants treated with 0.5 μM flg22 or 100 μM naringenin. Samples were collected 24 h post-treatment. Transcript levels of *PR* gene are increased in Col-0 plants. *PR1* **(C)** and *PR2* **(D)** transcript levels were measured by qPCR using specific primers. *Tubulin* was used as an internal control. Error bars indicate SD. Different letters above bars indicate statistically significant differences between samples, according to Tukey’s honestly significant difference test (*P* < 0.05). The experiment was repeated three times with similar results.

Flavonoids play roles in plant resistance against pathogenic bacteria and fungi through the induction of *PR* genes (Mierziak et al., 2014). For example, quercetin and its derivatives are known to induce fungal pathogen resistance (Parvez et al., 2004) and also induce bacterial pathogen resistance (Jia et al., 2010; Yang et al., 2016). To investigate whether naringenin has antimicrobial activity against *Pst* DC3000, we performed the pathogen growth assay on solid medium sprayed with different concentrations of naringenin. However, we could not find the antimicrobial activity of naringenin at all tested concentration (Supplementary Figure 1). Therefore, we hypothesized that naringenin could also induce pathogen resistance in plants. To test this hypothesis, we investigated the effects of naringenin on defense response against *Pst* DC3000. After inoculation with *Pst* DC3000, chlorotic symptoms were observed on the leaves without pretreated with naringenin at 3 dpi. However, attenuated disease symptoms were observed in leaves pretreated with naringenin. In addition, bacterial growth was reduced in leaves, which were pretreated with naringenin (Figures 1A,B). These results suggest that naringenin also induces pathogen resistance.

To examine whether naringenin induces basal pathogen resistance, the expression of *PR1* and *PR2* genes were analyzed after the treatment with naringenin by qPCR. As a positive control, a representative PAMP molecule, flg22 was treated. The expression levels of *PR1* and *PR2* genes by the treatment of flg22 were approximately 73.8-fold and 17.9-fold higher than the control, respectively. As expected, we also found that the expressions of *PR1* and *PR2* genes by the treatment with naringenin were approximately 46.1-fold and 9.5-fold higher than the control, respectively (Figures 1C,D). These results suggest that naringenin strongly induces the expression of *PR* genes.

### Naringenin Induces Resistance Through the Monomerization of NPR1

**FIGURE 2:**
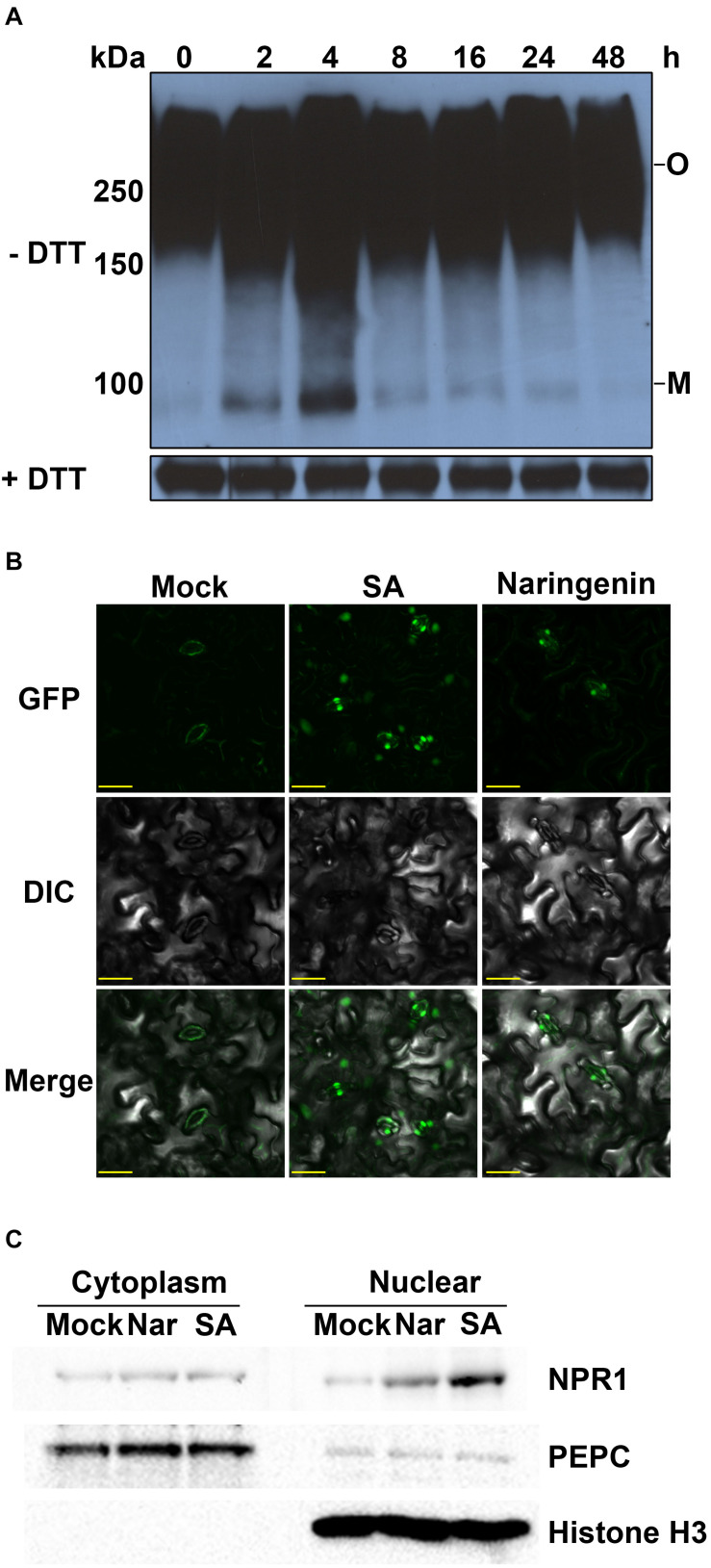
Naringenin induces the monomerization and nuclear translocation of NPR1. **(A)** The monomerization of NPR1 by naringenin. *35S::NPR1-GFP* transgenic plants (in *npr1-2*) were treated with 100 μM naringenin. Plants were collected at the indicated time points after naringenin treatment. Total protein (30 μg) was extracted and subjected to SDS-PAGE with or without DTT in the sample buffer (62.5 mM Tris-HCl pH 6.8, 10% glycerol, 1% lithium dodecyl sulfate (LDS), and 0.005% Bromophenol Blue) and analyzed using immunoblot using polyclonal anti-NPR1 antibodies. Both oligomeric (O) and monomeric (M) forms of NPR1 were detected. **(B)** Subcellular localization of NPR1-GFP by naringenin. Confocal microscope images of NPR1-GFP fluorescence in representative guard cells. Leaf tissues from the *35S::NPR1-GFP* treated with or without 100 μM naringenin for 3 h viewed with a fluorescence microscope. Scale bar represents 20 μm. The experiment was repeated three times with similar results. **(C)** Western blot analyses of NPR1 in nuclear and cytoplasmic fractions. Nuclear and cytoplasmic proteins were fractionated from total extracts from leaves of NPR1-GFP/npr1-2 after treatment with or without 100 μM of naringenin for 3 h. The blots were probed with an anti-NPR1 antibody. To determine the purity of fractions, anti-PEPC and Histone H3 antibodies were used for the Western blots of cytoplasmic and nuclear fractions, respectively.

The biological activity of NPR1 is enforced through a conformational change that rely on direct interaction with SA (Wu et al., 2012). The monomerization of NPR1 is required for the transcriptional activation of *PR* genes (Mou et al., 2003). Therefore, we hypothesized that naringenin induces conformational changes of NPR1. To test this hypothesis, a time-course experiment was performed to investigate the kinetics of NPR1 monomerization by naringenin. The monomerized NPR1 was detected under non-reducing conditions by Western blot. As reported, most of NPR1 was existed in oligomer forms not only in the absence but also in the presence of naringenin. Surprisingly, the monomer form of NPR1 was increased by the treatment with naringenin and peaks at 4 h after treatment (Figure 2A). This result suggests that naringenin induces the monomerization of NPR1.

It was well known that the nuclear translocation of NPR1 after monomerization is required for the induction of *PR* gene expressions (Kinkema et al., 2000; Maier et al., 2011). To investigate whether naringenin induces the nuclear localization of NPR1, we examined subcellular fluorescence of GFP tagged NPR1 in stomatal guard cells of NPR1-GFP/*npr1*-*2* plants after the treatment with naringenin. As a result, almost NPR1-GFP was detected in the cytoplasm of guard cells before the treatment. However, NPR1-GFP was strongly detected in the nucleus of guard cells at 3 h after naringenin treatment (Figure 2B). This result strongly suggests that naringenin induces the nuclear translocation of NPR1. To verify that naringenin induces the nuclear translocation of NPR1, we performed nuclear fractionation in NPR1-GFP/*npr1-2* after treatment with naringenin. As a result, we found that the nuclear translocation of NPR1 was obviously increased by the treatment with naringenin or SA (Figure 2C). Taken together, these data indicate that naringenin increases the nuclear translocation of NPR1 through monomerization.

To explore whether NPR1 plays a crucial role in naringenin-induced resistance, we investigated the effects of naringenin on pathogen resistance against *Pst* DC3000 in *npr1-1* plant. As a result, naringenin significantly reduced bacterial growth in Col-0 but not in *npr1-1* (Supplementary Figure 2). Consistently, the induction of *PR1* gene expression by naringenin was also impaired in *npr1-1* (Supplementary Figure 3). These data indicate that NPR1 is required for the naringenin-induced pathogen resistance.

### Naringenin Induces SA Biosynthesis-Related Genes and SA Accumulation

**FIGURE 3:**
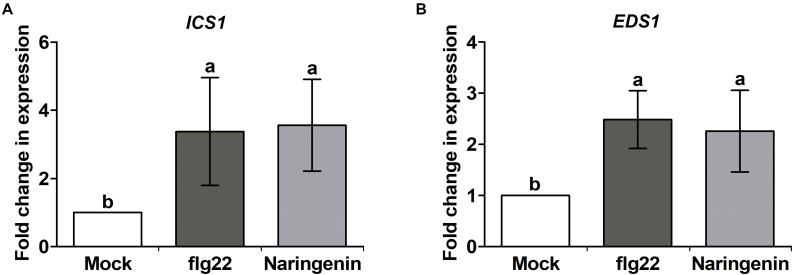
Naringenin increases expression of SA biosynthesis-related genes. Expression of SA biosynthesis-related genes by naringenin. Total RNA was extracted from Col-0 plants treated 0.5 μM flg22 or 100 μM naringenin. Samples were collected 24 h post-treatment. Transcript levels of SA biosynthesis-related genes are increased in Col-0 plants. *ICS1* **(A)** and *EDS1* **(B)** transcript levels were measured by qPCR using specific primers. *Tubulin* was used as an internal control. Error bars indicate SD. Different letters above bars indicate statistically significant differences between samples, according to Tukey’s honestly significant difference test (*P* < 0.05). The experiment was repeated three times with similar results.

**FIGURE 4:**
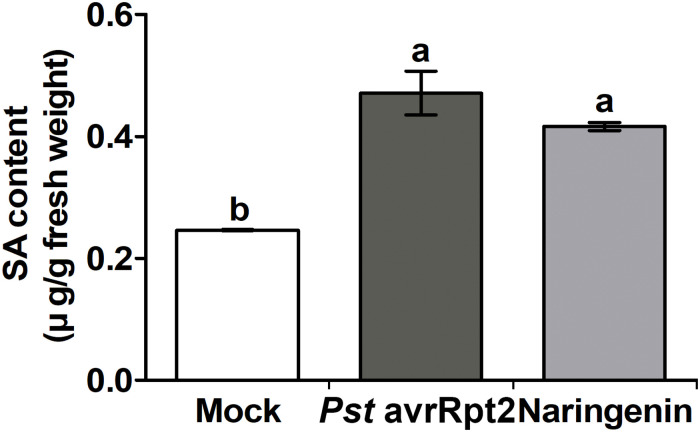
Naringenin induces the accumulation of SA. Accumulation of SA by naringenin. SA was extracted from Col-0 plants treated with *Pst* DC3000 *avrRpt2* or 100 μM naringenin. The leaves were sampled 24 h after treatment. Accumulation of SA was measured by HPLC-MS/MS. Data represent the mean SD of at least three biological replicate leaf samples from different plants. Each biological replicate consists of three leaves from three plants.

SA accumulation is closely associated with resistance against biotrophic and hemibiotrophic bacterial pathogens (van Butselaar and Van den Ackerveken, 2020). Previously, it was reported that other flavonoids-mediated pathogen resistance was activated by the SA-dependent pathway (Jia et al., 2010; Yang et al., 2016). To test whether SA biosynthesis-related genes could be induced by naringenin, the expressions of representative SA biosynthesis-related genes, *EDS1*, *ICS1* and *PAL1* were measured by using qPCR. The expressions of *EDS1* and *ICS1* genes were increased approximately 2.2-fold and 3.5-fold by the treatment with naringenin (Figure 3), whereas *PAL1* gene was not (Supplementary Figure 4). This result suggests that naringenin increases SA biosynthesis by isochorismate dependent pathway. The increased SA biosynthesis-related genes by naringenin prompted us to examine whether naringenin increases the level of SA. Therefore, we measured free SA amount in plant after the treatment with naringenin. Interestingly, the levels of free SA were 2-fold increased by naringenin (Figure 4). This result indicates that naringenin induces the accumulation of SA in plants.

### Naringenin Induces the Accumulation of ROS

**FIGURE 5:**
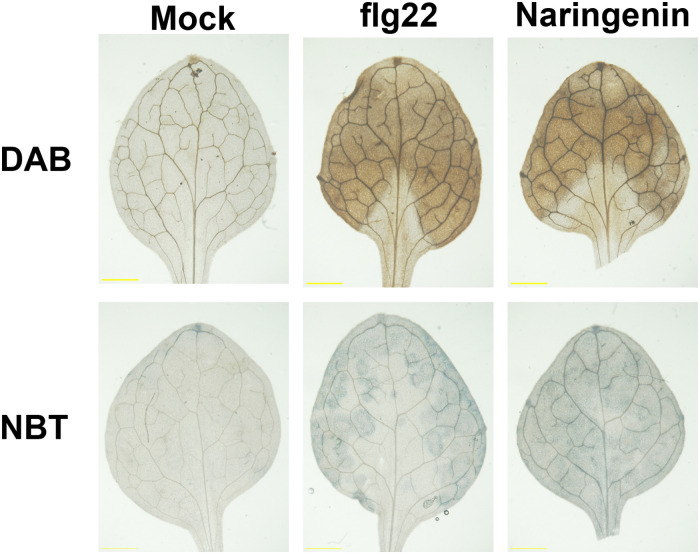
ROS is accumulated by naringenin. H_2_O_2_ and O_2_^–^ accumulation was visualized in Col-0 leaves after 0.5 μM flg22 or 100 μM naringenin treatment. The leaves were sampled at 24 h after treatments and stained with DAB and NBT. Scale bar represents 1 mm. The experiment was repeated three times with similar results.

The infections of most pathogens show oxidative burst, which constitutes the production of ROS, including superoxide anion (O_2_^–^) and hydrogen peroxide (H_2_O_2_). The increased H_2_O_2_ level contributes to the upregulation of genes associated with defense responses in plants (Apel and Hirt, 2004). Flavonoids are known to function as antioxidant agents by scavenging reactive oxygen species (Treml and Šmejkal, 2016). However, a previous study reported that quercetin activated defensive responses via H_2_O_2_ burst in *Pst* DC3000-challenged *Arabidopsis* (Jia et al., 2010). In order to test whether ROS is accumulated by the treatment with naringenin, we performed histochemical staining with 3, 3′-diaminobenzidine (DAB) and nitro blue tetrazolium (NBT) to monitor the production of H_2_O_2_ and O_2_^–^, respectively. As expected, the accumulation of H_2_O_2_ and O_2_^–^ were clearly detected by treatment with flg22. The accumulation of H_2_O_2_ and O_2_^–^ were also detected by the treatment with naringenin (Figure 5), indicating that naringenin induces ROS burst in plants.

### MPK3 and MPK6 Are Required for Naringenin-Induced Resistance

**FIGURE 6:**
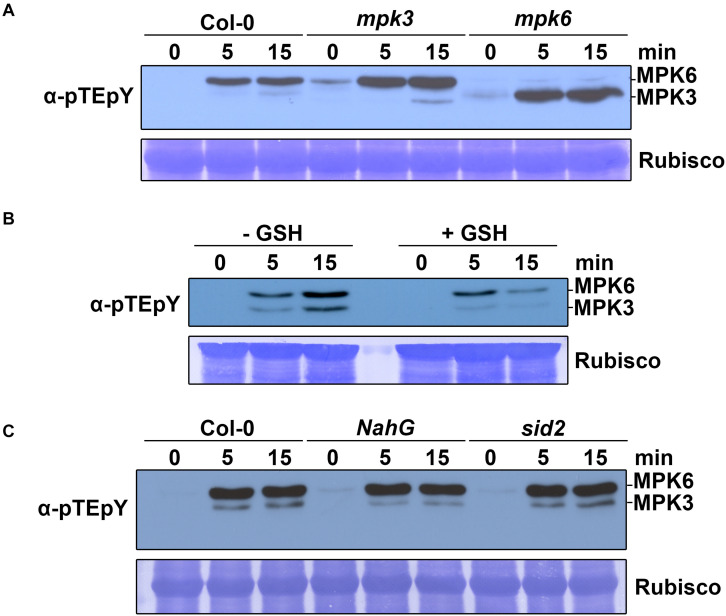
Naringenin activates MPK3 and MPK6. **(A)** Activation of MAPK by naringenin. Two-week-old Col-0, *mpk3* and *mpk6* plants were treated with 100 μM naringenin. Samples were collected at 0, 5, and 15 min after treatment. Total protein extracts were prepared from those treated plants. MAPK activation was detected by immunoblotting with anti-p44/42 antibodies (Cell Signaling Technology, United States). Input was visualized by Rubisco. The experiment was performed three times with similar results. **(B)** ROS-dependent activation of MAPK by naringenin. Two-week-old Col-0 plants were pretreated for 1 h with or without 200 μM GSH and subsequently treated with 100 μM naringenin and then analyzed as detailed in above. **(C)** SA-independent activation of MAPK by naringenin. Two-week-old Col-0, *NahG* and *sid2* plants were treated with 100 μM naringenin and then analyzed as detailed in above. The experiment was repeated three times with similar results.

The activation of MAPK cascades is one of the well-known earliest defense signaling against pathogen attack (Meng and Zhang, 2013). To monitor whether MAPKs were activated by naringenin, we measured the MAPK activity by immunoblotting using dual TEY phosphorylation antibodies, that recognize active forms of MAPKs (Beckers et al., 2009; Wang et al., 2018). Protein was extracted from two-week-old plants at 0, 5 and 15 min following naringenin treatment. As a result, both MPK3 and MPK6 were activated by naringenin in Col-0 plants. In *mpk3* and *mpk6* mutant plants, naringenin could not activate the missing kinase and confirm the identity of the bands detected with dual TEY phosphorylation antibodies. The MAPK activity was strongest at the 15 min time point after treatment (Figure 6A). Therefore, we concluded that naringenin activates MPK3 and MPK6 in *Arabidopsis*.

MAPKs were believed to function downstream of early ROS burst in plant immunity. ROS are known to be associated with the activation of defense-related MAPK in *Arabidopsis* (Meng and Zhang, 2013). To determine whether ROS also plays a role in naringenin-induced MPK3 and MPK6 activation, we examined the activation of MAPK by naringenin after pretreated with or without glutathione (GSH). GSH is widely used as a general ROS scavenger to reduce the level of ROS (Liu et al., 2010; Ramírez et al., 2013). The activation of MPK3 and MPK6 by naringenin was significantly decreased by the pre-treatment with GSH (Figure 6B), suggests that naringenin activates MPK3 and MPK6 through the accumulation of ROS. To test whether SA is associated with naringenin-induced MPK3 and MPK6 activation, we measured activation of MAPK by naringenin in *sid2* mutant, SA deficient mutant and *NahG* transgenic plants, SA non-accumulation plant. The naringenin-induced activations of MPK3 and MPK6 were also similarly observed in *sid2* and *NahG* (Figure 6C), suggests that naringenin SA-independently activates MPK3 and MPK6.

**FIGURE 7:**
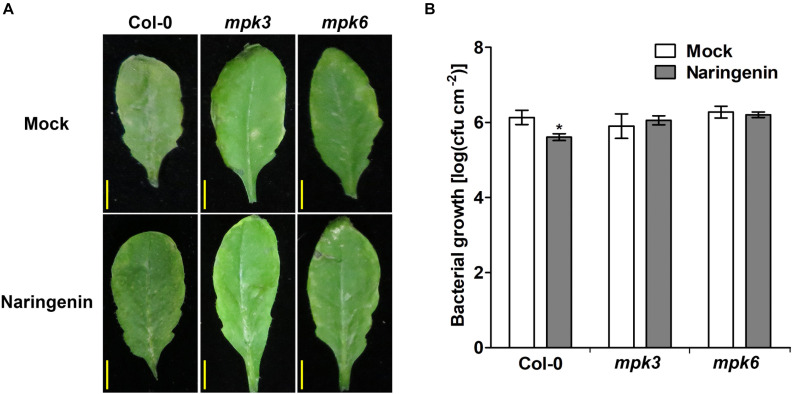
Pathogen resistance by naringenin is compromised in *mpk3* and *mpk6* mutants. **(A)** Disease symptoms of *Pst* DC3000-treated Col-0, *mpk3* and *mpk6* mutant in the absence and presence of naringenin. **(B)** The growth of *Pst* DC3000 in control or in naringenin-pretreated Col-0, *mpk3* and *mpk6* mutant plants. Details as described in Figure 1. Scale bar represents 0.5 cm.

To test whether MAPKs are involved in naringenin-induced pathogen resistance, we investigated the effects of naringenin on bacterial resistance in *mpk3* and *mpk6* plants. As a result, naringenin induced pathogen resistance in Col-0 but not in *mpk3* and *mpk6* (Figure 7). These results indicate that MPK3 and MPK6 are required for naringenin-induced pathogen resistance.

### Naringenin Induces the Monomerization of NPR1 Through MAPK

**FIGURE 8:**
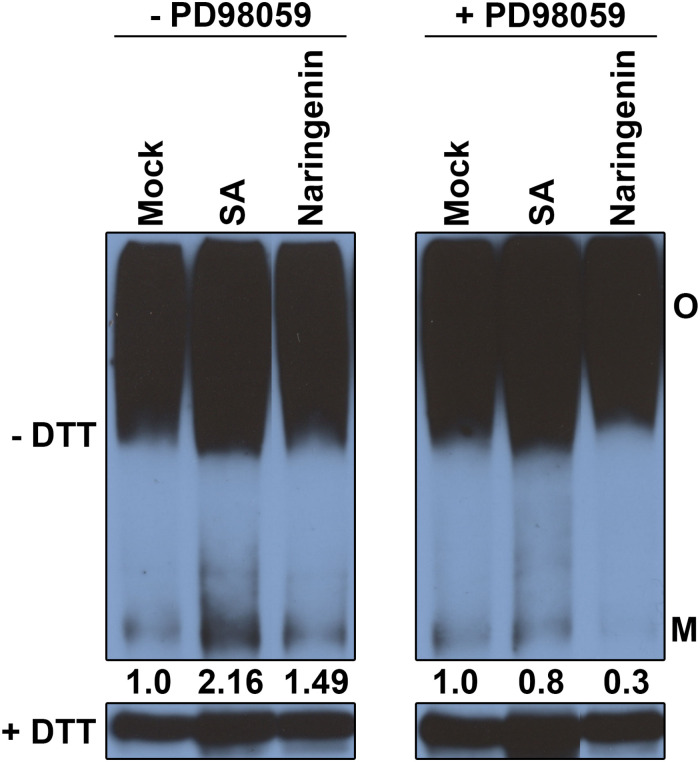
The monomerization of NPR1 by naringenin requires MAPK activity. *35S:NPR1-GFP* transgenic plants (in *npr1-2*) were pretreated with or without PD98059 (50 μM) and then subsequently treated with 200 μM SA or 100 μM naringenin. Plants were collected at 4 h after treatment. Conformation of NPR1 after naringenin induction was detected by immunoblot under non-reducing conditions. Total protein (30 μg) was extracted and subjected to SDS-PAGE with or without DTT in the sample buffer (62.5 mM Tris-HCl pH 6.8, 10% glycerol, 1% LDS, and 0.005% Bromophenol Blue) and analyzed using immunoblot using polyclonal anti-NPR1 antibodies. Both oligomeric (O) and monomeric (M) forms of NPR1-GFP were detected. The experiment was repeated three times with similar results.

MPK6 is known to be involved in the monomerization of NPR1 in the process of SA-induced leaf senescence (Chai et al., 2014). Therefore, we hypothesize that a MAPK cascade is involved in naringenin-induced monomerization of NPR1. To test this hypothesis, naringenin-induced monomerization of NPR1 was examined after pre-treatment with PD98059, the inhibitor of MAP kinase kinases. The monomer form of NPR1 was measured by Western blot using anti-NPR1 antibodies. Expectedly, naringenin induces the increase of NPR1 monomer in the absence of PD98059. However, the amount of NPR1 monomer induced by naringenin was significantly decreased in the presence of PD98059 (Figure 8). These results indicated that naringenin induces the monomerization of NPR1 through the activation of MAPK.

### SnRK2.8 Is Phosphorylated by MPK3

It was reported that the phosphorylation of NPR1 by SnRK2.8 partially contributes to the monomerization of NPR1 (Lee et al., 2015). However, the upstream component in pathogen resistance signaling pathway that regulates the activity of SnRK2.8 was not identified yet. Here, we suspected that SnRK2.8 is a target of MAPKs because it contains two conserved potential phosphorylation sites of MAPKs. To test this possibility, we performed *in vitro* kinase assays with His tagged MAPKs and GST tagged SnRK2.8. As a result, we found that SnRK2.8 was phosphorylated by MPK3 but not by MPK4 and MPK6 (Supplementary Figure 5). This result suggests that SnRK2.8 may be involved in the monomerization of NPR1 by naringenin-activated MPKs.

## Discussion

### Naringenin Is a Flavonoid That Induces Pathogen Resistance

As a flavonoid, naringenin is well known to have various biological functions such as UV protectants, ROS scavengers, anti-inflammatory agents and anti-cancer agents like other flavonoids (Brunetti et al., 2013; Venkateswara Rao et al., 2017; Henry-Kirk et al., 2018). Previously, flavonoids including naringenin are accumulated after infection with biotrophic pathogen *Plasmodiophora brassicae, Pseudomonas siringae pv. Pisi* and *X. campestris pv. Malvacearum* (Beckman, 2000; Kangatharalingam et al., 2002; Päsold et al., 2010; Makarova et al., 2016). Furthermore, we found that naringenin induces pathogen resistance to *Pst* DC3000 (Figures 1A,B), suggesting that accumulated naringenin by pathogen leads to pathogen resistance (Jia et al., 2010; Yang et al., 2016). However, the molecular mechanism of how naringenin induces pathogen resistance is unknown. In this study, we also found that H_2_O_2_ level and *PRs* transcripts were increased by treatment with naringenin (Figures 1C,D, 5). Similarly, SA, azelaic acid, pipecolic acid, thiamine and riboflavin confer pathogen resistance through ROS accumulation and increased expression of *PR* genes (Ahn et al., 2005; Jung et al., 2009; Zhang et al., 2009; Bernsdorff et al., 2016). These results suggest that naringenin induces plant pathogen resistance by similar mechanisms with other resistance inducing molecules.

### Naringenin May Act as a Prooxidant in Pathogen Resistance

Flavonoids are well known as potent antioxidants and ROS scavengers *in vitro* (Rice-Evans, 2001). In addition, flavonoids are reported to decrease ROS levels by inhibiting prooxidant enzymes, cyclooxygenase and lipoxygenase (Glazebrook et al., 1996). However, flavonoids and carotenoids can also act as a prooxidant at physiological pH just likely that polyphenol, an antioxidant, can act as a prooxidant in the presence of metal ions (Guardado et al., 2012; Eghbaliferiz and Iranshahi, 2016). In this study, we showed that naringenin induces the accumulation of ROS (Figure 5), which suggests that naringenin can act as a prooxidant. Consistently, naringenin has prooxidant activity in human lymphocytes (Yen et al., 2003), and naringenin does not show antioxidant activity even naringenin is highly accumulated in roots (Päsold et al., 2010). In addition, quercetin, a different flavonoid, also induces H_2_O_2_ to result in pathogen resistance (Jia et al., 2010). Although quercetin is proposed to induce oxidative stress indirectly through inhibition of nitric oxide dioxygenase in tumor cells (Scheit and Bauer, 2015), it is still unknown how flavonoids act as prooxidants. Further studies are required for the detailed molecular mechanisms by which flavonoids increase ROS production.

### MPK3 and MPK6 Are Required for the Naringenin-Induced Pathogen Resistance

MPK3 and MPK6 are the representative positive regulator of immune responses including defense gene activation, ROS generation, hypersensitive response, biosynthesis of camalexin and biosynthesis of ethylene (Meng and Zhang, 2013; Zhang et al., 2018). In this study, we showed that naringenin increased not only the activity of MPK3 and MPK6 (Figure 6) but also the monomerization of NPR1 through activated MAPK (Figure 8). In contrast, the induction of pathogen resistance by naringenin was not shown in *mpk3* and *mpk6* (Figure 7). This observation is similar to a previous report that BTH, riboflavin and pipecolic acid induce pathogen resistance by increasing the activity of MPK3 and MPK6 (Nie and Xu, 2016; Wang et al., 2018). These results suggest that naringenin also induces pathogen resistance through the activation of MAPK pathways. Since the levels of ROS and SA were increased by treatment with naringenin in plants, we speculate that naringenin activates MAPK by the increase of ROS and SA.

### Naringenin Induces Pathogen Resistance by the Activation of NPR1

Plant pathogen resistance is obtained by the rapid induction of immune responses by SA (Ding and Ding, 2020). SA-mediated immune responses are reported to be activated by the coordination of different NPR proteins (Backer et al., 2019). It has been reported that pathogen resistance inducing molecules confer pathogen resistance in NPR1 dependent manner (Nie and Xu, 2016; Yang et al., 2016). In this study, we showed that naringenin not only increased SA level by induction of SA biosynthetic gene (Figures 3, 4) but also induced nuclear translocation of NPR1 (Figure 2). In addition, naringenin-induced pathogen resistance was abolished in npr1-1 mutant (Supplementary Figure 2). These results suggest that naringenin induces pathogen resistance through the activation of NPR1. The molecular mechanism to explain how naringenin activates NPR1 should be elucidated in further study.

**FIGURE 9:**
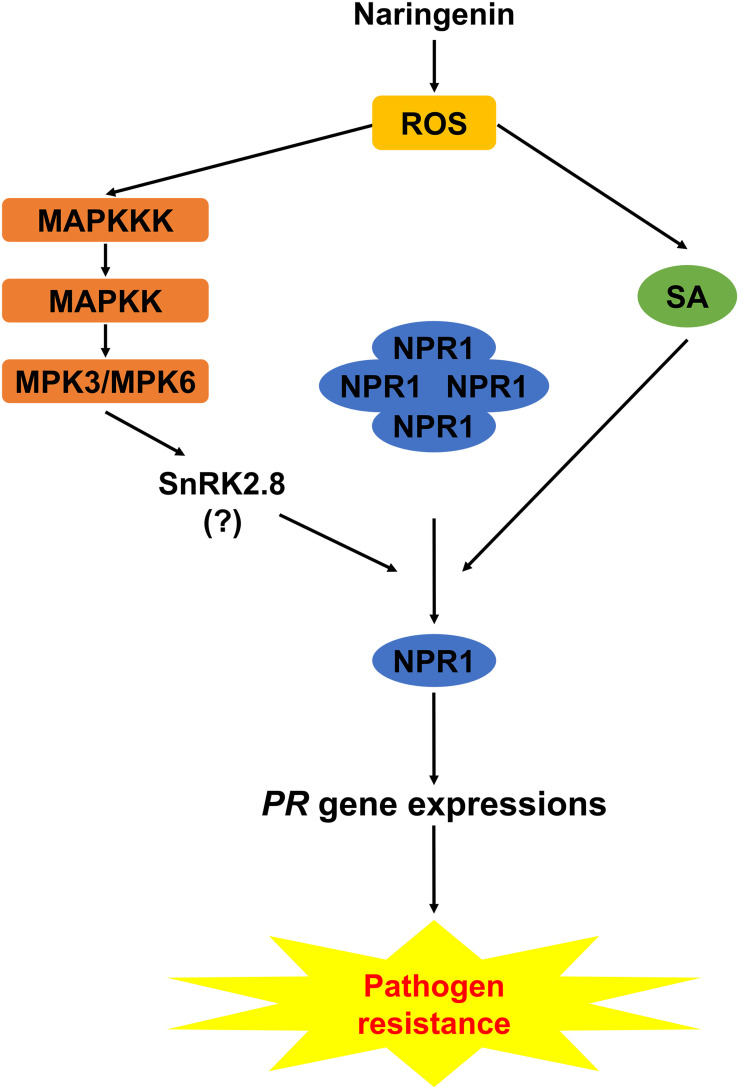
Working model explaining how naringenin induces pathogen resistance. Naringenin activates the production of ROS in plants. In SA-dependent pathway, naringenin activates the monomerization and nuclear translocation of NPR1 by the accumulation of SA. In SA-independent pathway, naringenin activates MPKs, and the activated MPKs possibly activate SnRK2.8 that promotes the monomerization and nuclear translocation of NPR1.

Based on this study, we proposed the working model explaining how naringenin induces pathogen resistance (Figure 9). In this model, both the SA-dependent and the SA-independent pathways in the activation of NPR1 by naringenin were suggested. In the SA-dependent pathway, naringenin activates the nuclear translocation and monomerization of NPR1 by the accumulation of SA that induces a thioredoxin-mediated reduction of NPR1. In the SA-independent pathway, naringenin activates MPKs, and then the activated MPKs possibly activate SnRK2.8 which promotes the phosphorylation and monomerization of NPR1 (Chai et al., 2014; Lee et al., 2015). In this study, we have shown that MPK3 phosphorylates SnRK2.8 (Supplementary Figure 5). However, we have detected the phosphorylation of NPR1 by neither unphosphorylated nor phosphorylated SnRK2.8 by MPKs at our experimental condition. The further study identifying how the SA-independent pathway contributes to naringenin-mediated activation of NPR1 should be done in the future.

## Data Availability Statement

The original contributions presented in the study are included in the article/Supplementary Material, further inquiries can be directed to the corresponding author/s.

## Author Contributions

JA and WC designed and planned the experiments. JA, UV, NN, and HD performed the experiments. JA, SB, and SuK analyzed the data. JA, SaK, and WC wrote the article. All authors contributed to the article and approved the submitted version.

## Conflict of Interest

The authors declare that the research was conducted in the absence of any commercial or financial relationships that could be construed as a potential conflict of interest.
