# Supplementary material for: Naringenin Induces Pathogen Resistance Against Pseudomonas syringae Through the Activation of NPR1 in Arabidopsis
Source: Front Plant Sci. 2021 May 20;12:672552. doi: 10.3389/fpls.2021.672552 (PMC8173199; doi:10.3389/fpls.2021.672552)
Supplement: Supplementary file 1 [file Data_Sheet_1.docx]

**
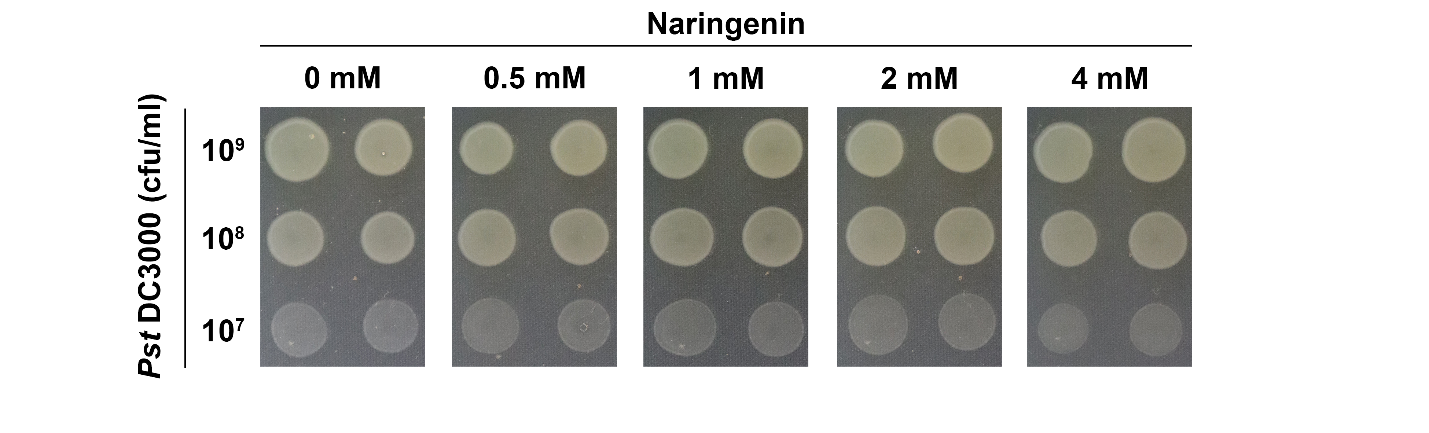
**

**Supplementary Figure 1.** Measurement of antimicrobial activity of naringenin against *Pst* DC3000. *Pst* DC3000 was cultured on KB medium after the sprays of 0 to 4 mM naringenin. The photographs were taken after 24 h incubation at 28℃.


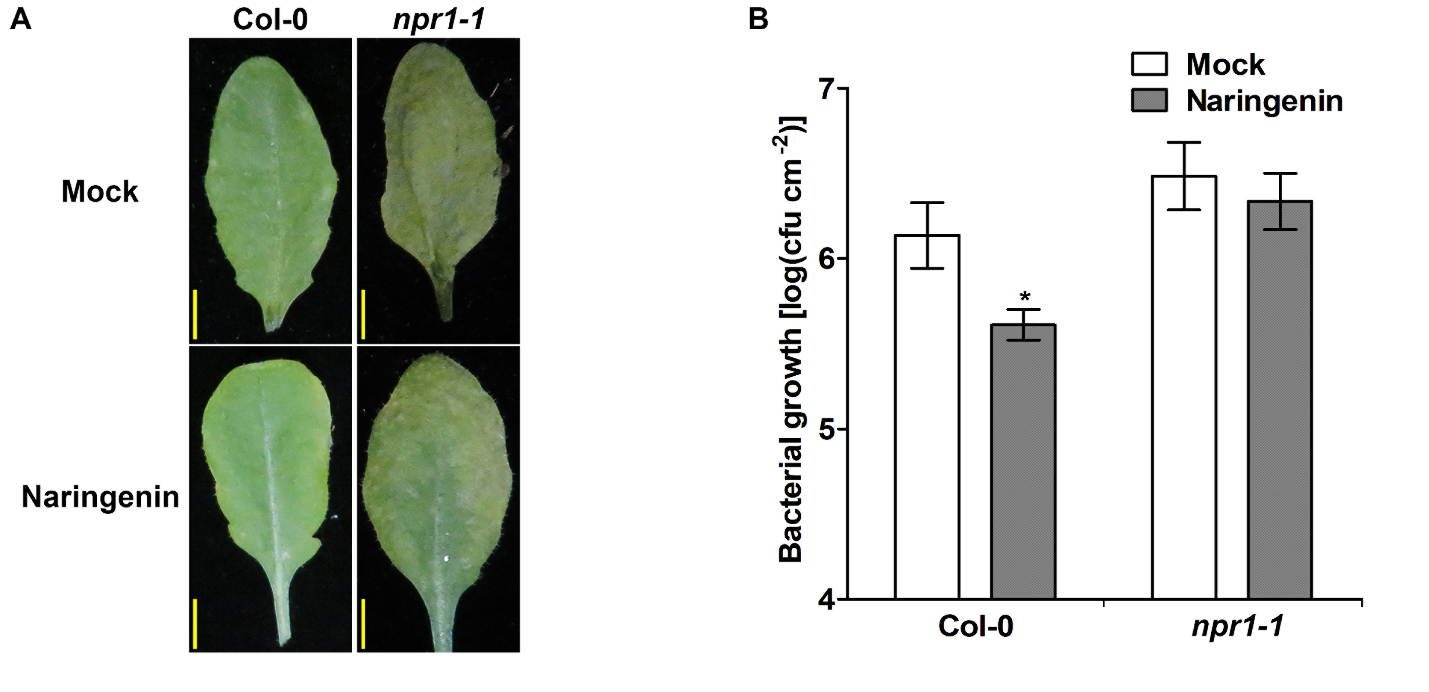


**Supplementary Figure 2** Pathogen resistance by naringenin is compromised in *npr1-1* mutant. **(A)** Disease symptoms of *Pst* DC3000-treated Col-0 and *npr1-1* mutant in the absence and presence of naringenin. **(B)** The growth of *Pst* DC3000 in control or in naringenin-pretreated Col-0 and *npr1-1* mutant plants. Scale bar represents 0.5 cm. Details as described in Figure 1.


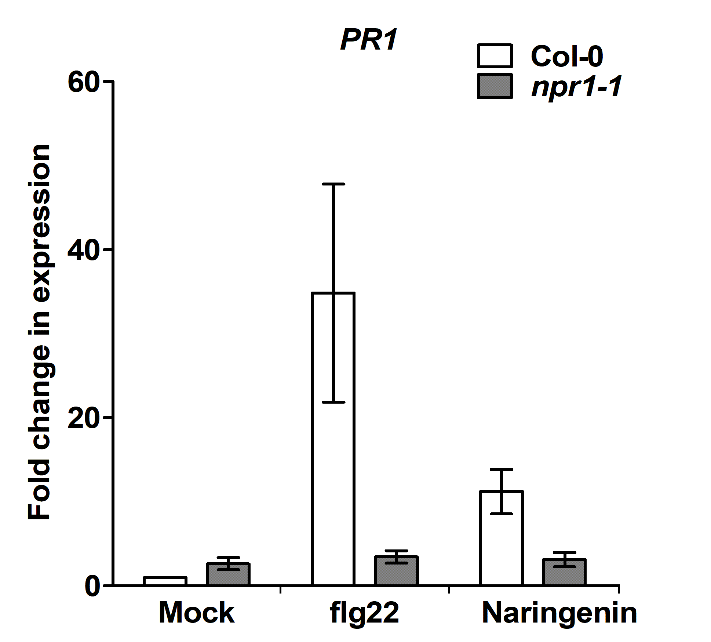


**Supplementary Figure 3** *PR1* gene expression by naringenin is compromised in *npr1-1* mutant. Total RNA was extracted from Col-0 and *npr1-1* mutant plants treated with 0.5 μM flg22 or 100 μM naringenin. Transcript levels of *PR1* gene are increased in Col-0 and *npr1* mutant plants. *PR1* transcript levels were measured by qPCR using specific primers. Error bars indicate SD (n= 3 biological replicates). Bars marked with different letters are significantly different (P < 0.05).


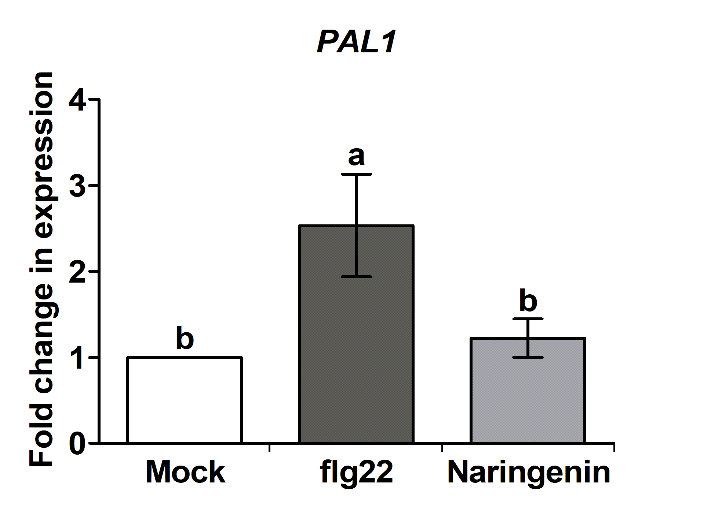


**Supplementary Figure 4** The transcript level of *PAL1* genes in response to naringenin. Total RNA was extracted from Col-0 plants treated with 0.5 μM flg22 or 100 μM naringenin for 24 h. *PAL1* transcript levels were measured by qPCR using specific primers. *Tubulin* was used as an internal control. Error bars indicate SD. Different letters above bars indicate statistically significant differences between samples, according to Tukey’s honestly significant difference test (P < 0.05). The experiment was repeated three times with similar results.


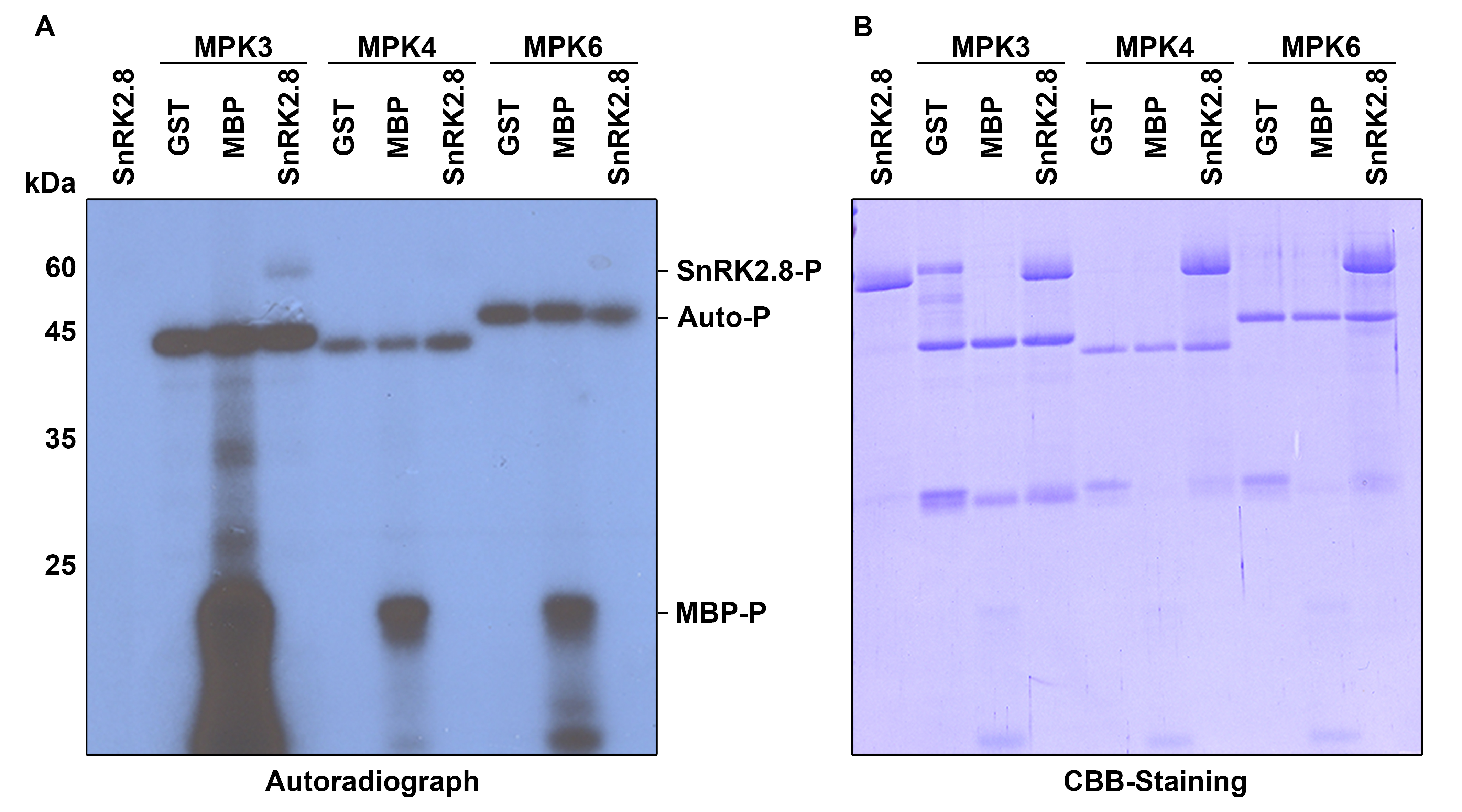


**Supplementary Figure 5** SnRK2.8 is phosphorylated by MPK3. Recombinant proteins of the GST-tagged SnRK2.8, His-tagged MPK3, -4 and -6 were purified and subjected for *in vitro* kinase assay. Recombinant proteins were separated by 10% SDS-PAGE after incubation in protein kinase buffer containing [γ-^32^P] ATP. Phosphorylated SnRK2.8 was detected by autoradiography after gel electrophoresis **(A)**. Recombinant MPK3, -4, -6 and SnRK2.8 were detected by Coomassie brilliant blue (CBB) staining **(B)**. MBP and GST were used as positive and negative control substrates, respectively.

**Supplementary Table 1** Primers used for quantitative PCR.

| **Construct** | **Position** | **Sequence** |
| --- | --- | --- |
| **PR1** | **F** | 5'- GTGGGTTAGCGAGAAGGCTA -3' |
|  | **R** | 5'- ACTTTGGCACATCCGAGTCT -3' |
| **PR2** | **F** | 5'- CGGTACATCAACGTTGGAA -3' |
|  | **R** | 5'- GCGTAGTCTAGATGGATGTT -3' |
| **ICS1** | **F** | 5'- AGTGAATTTGCAGTCAGTCGGGAT -3' |
|  | **R** | 5'- AATCGCCTGTAGAGATGTTGT -3' |
| **EDS1** | **F** | 5'- TCGAAGGGGACATAGATTGG -3' |
|  | **R** | 5'- CTTTTCATGTACGGCCCTGT -3' |
| **Tubulin** | **F** | 5'- CCAACAACGTGAAATCGACAG -3' |
|  | **R** | 5'- TCTTGGTATTGCTGGTACTCT -3' |
| **PAL1** | **F** | 5’-TGTAGCGCAACGTACC -3’ |
|  | **R** | 5'-GTTCGGGATAGCCGATG-3’ |

**Supplementary Table 2** Primers used for construction of *SnRK2.8*, *MPK3*, *MPK4* and *MPK6* plasmids.

| **Construct** | **Position** | **Sequence** |
| --- | --- | --- |
| **GST-SnRK2.8** | **F** | 5'- ggatccATGGAGAGGTACGAAATAGT -3' |
|  | **R** | 5'- ctcgagTCACAAAGGGGAAAGGAGAT-3' |
| **His-MPK3** | **F** | 5'- ggatccATGAACACCGGCGGTGGCCA -3' |
|  | **R** | 5'- gtcgacCTAACCGTATGTTGGATTGA -3' |
| **His-MPK4** | **F** | 5'- ggatccATGTCGGCGGAGAGTTGTTT -3' |
|  | **R** | 5'- gtcgacCTACACTGAGTCTTGAGGAT -3' |
| **His-MPK6** | **F** | 5'- ggatccATGGACGGTGGTTCAGGTCA -3' |
|  | **R** | 5'- gtcgacCTATTGCTGATATTCTGGAT -3' |
